# Tentative Identification of the Second Substrate Binding Site in Arabidopsis Phytochelatin Synthase

**DOI:** 10.1371/journal.pone.0082675

**Published:** 2013-12-05

**Authors:** Ju-Chen Chia, Chien-Chih Yang, Yu-Ting Sui, Shin-Yu Lin, Rong-Huay Juang

**Affiliations:** Department of Biochemical Science and Technology, College of Life Science, National Taiwan University, Taipei, Taiwan; Wake Forest University, United States of America

## Abstract

Phytochelatin synthase (PCS) uses the substrates glutathione (GSH, γGlu-Cys-Gly) and a cadmium (Cd)-bound GSH (Cd∙GS_2_) to produce the shortest phytochelatin product (PC_2_, (γGlu-Cys)_2_-Gly) through a ping-pong mechanism. The binding of the 2 substrates to the active site, particularly the second substrate binding site, is not well-understood. In this study, we generated a structural model of the catalytic domain of Arabidopsis AtPCS1 (residues 12–218) by using the crystal structure of the γGlu-Cys acyl-enzyme complex of the PCS of the cyanobacterium *Nostoc* (NsPCS) as a template. The modeled AtPCS1 revealed a cavity in proximity to the first substrate binding site, consisting of 3 loops containing several conserved amino acids including Arg152, Lys185, and Tyr55. Substitutions of these amino acids (R152K, K185R, or double mutation) resulted in the abrogation of enzyme activity, indicating that the arrangement of these 2 positive charges is crucial for the binding of the second substrate. Recombinant AtPCS1s with mutations at Tyr55 showed lower catalytic activities because of reduced affinity (3-fold for Y55W) for the Cd∙GS_2_, further suggesting the role of the cation-π interaction in recognition of the second substrate. Our study results indicate the mechanism for second substrate recognition in PCS. The integrated catalytic mechanism of PCS is further discussed.

## Introduction

Phytochelatins (PC) are (γGlu-Cys)_n_-Gly (n = 2–11) polymers representing major detoxification components in plants, fungi, and other organisms [1-4]. These cysteine (Cys)-rich polypeptides act as high-affinity metal chelators [5,6] and facilitate the vacuolar sequestration of heavy metals [7-10]. Phytochelatin synthase (PCS; EC 2.3.2.15) is a γ-glutamylcysteine dipeptidyl transpeptidase that catalyzes the synthesis of PC using glutathione (GSH, γGlu-Cys-Gly) or previously synthesized PC as substrates [11]. Previous studies have identified and characterized the genes encoding PCS in various eukaryotic organisms, such as *Arabidopsis thaliana* (*AtPCS1*) [12,13], *Triticum aestivum* (*TaPCS1*) [14], *Lotus japonicas* (*LjPCS1*) [15], *Schizosaccharomyces pombe* (*SpPCS*) [12,14], the nematode *Caenorhabditis elegans* (*CePCS1*) [16], and the cadmium hyperaccumulator *Thlaspi caerulescens* (*TcPCS1*) [17]. Studies have also identified a gene encoding a PCS-like protein in the genome of the cyanobacterium *Nostoc* [18,19]. PCS is constitutively expressed at a transcriptional level, and cadmium (Cd) treatment marginally upregulates this expression [20,21]. Plants synthesize PC on exposure to heavy metals, indicating that metal ions immediately activate PCS catalysis [2,3]. As shown in previous studies, a variety of heavy metals can activate the PCS proteins [22,23], and PCS is posttranslationally regulated by metal ions [20]. Heavy metals might bind directly to several Cys-rich motifs in PCS, resulting in augmentative activation [24-27]. In our previous study, we showed that AtPCS1 is activated by protein phosphorylation in the presence of Cd, providing further evidence of the posttranslational regulation of PCS [28]. 

Several studies have shown the catalytic activation of PCS by heavy metals [20,25,29,30]. The eukaryotic PCS proteins are 50–55 kDa polypeptides that display 40%–50% sequence similarity and contain a highly conserved N-terminal domain, with a papain-like catalytic triad [25,27,31] and a variable C-terminal domain [10]. For example, in AtPCS1, the N-terminal domain contains the catalytic triad Cys 56, His 162, and Asp 180, and is responsible for the deglycylation of GSH and PC synthesis [25]. The truncated protein expressing the N-terminal domain still possesses its PC synthesis activity [29]. However, loss of the C-terminal region in this truncated protein substantially decreases its thermal stability and impairs the phytochelatin formation activity when certain heavy metals are employed in the assay (e.g. mercury and zinc, but not cadmium or copper) [29]. These characteristics indicate that the C-terminal domain is crucial for protein stability and for the recognition of heavy metals [27,29]. 

Vatamaniuk et al. first reported a mechanism for PC synthesis [20]. In standard PCS assay conditions, in which GSH occurs at a considerably higher level (millimolar) than Cd ions (micromolar), >98% of the total Cd added to the reaction medium is associated with GSH as bis(glutathionato)cadmium (Cd∙GS_2_), and the free Cd concentration is extremely low. Thus, GSH and Cd∙GS_2_ participate in the reaction catalyzed by AtPCS1 as 2 separate substrates. The synthesis of PC occurs through a substituted enzyme mechanism in 2 stages. First, the removal of Gly on the first GSH by PCS results in the formation of the γGlu-Cys acyl-enzyme intermediate. This γGlu-Cys unit is then transferred to Cd∙GS_2_ on the second substrate binding site to generate a simple PC [20,30,32]. Both GSH and its metal thiolate are required for maximal synthetic activity. Although the equilibrium dialysis for AtPCS1 indicates that 7 Cd ions are bound to the protein, heavy metals are considered to activate PCS primarily through the formation of the low *K*_m_ Cd∙GS_2_ substrates [20,30]. However, *S*-substituted GSH derivatives (e.g., *S*-methylglutathione) can substitute for both substrates to overcome the requirement of the enzyme for the heavy metals, suggesting that the decisive factor for core catalysis is the provision of GSH-like substrates containing blocked thiol groups [20,25,27].

Although previous studies have described the core catalytic mechanism of AtPCS1, the manner in which GSH and Cd∙GS_2_ interact with the enzyme is not well-understood. The crystal structure of a prokaryotic counterpart of PCS from the cyanobacterium *Nostoc* sp. PCC 7120 (NsPCS) provides local structural information on the catalytic site and its interacting molecules, and also shows that PCS is a dimer [31]. NsPCS is absent of the C-terminal domain and catalyzes the deglycylation of GSH to γGlu-Cys at a high rate, and the synthesis of PC_2_ at a relatively low rate, compared with the eukaryotic enzymes [18,19,33]. Detail structure analysis of NsPCS could explain why it might act as a hydrolase and weakly as a peptide ligase. The protein structure reveals a cavity in proximity to the first GSH binding site, which is proposed as the putative second GSH binding site [31]. Several conserved amino acid residues surrounding this site might be involved in the formation of the cavity and stabilize the binding GSH [31]. Because the amino acid sequence of NsPCS shows 36% identity with the N-terminal region of the AtPCS1, the NsPCS crystal structure can provide a template for the model simulation of the N-terminal domain of AtPCS1. This model can facilitate the more detailed investigation into the binding of the second substrate to PCS. 

## Materials and Methods

### AtPCS1 molecular modeling

The molecular model of AtPCS1 (residues12–218) and AtPCS1-R152K-K185R was generated using the coordinates of the *Nostoc* PCS structure (2BU3) as the template in Discovery Studio 3.0 (Accelrys) as described previously [28]. The model was subjected to energy minimization and validated using the Protein Health module within the Discovery Studio package. The substrate γ-glutamylcysteine was transferred into the model from the template crystal structure. The CDOCK method in the Discovery Studio package was used to identify the interaction of the ligand molecule with the second binding site of the protein.

### Site-directed mutagenesis of AtPCS1

The site-directed mutagenesis of AtPCS1 was performed directly on the pET28b-AtPCS1 vector using a QuickChange II Site-Directed Mutagenesis Kit (Agilent). The mutagenic oligonucleotides were designed to individually substitute the Gln50, Ser51, Glu52, Tyr55, Cys56, Arg152, Lys156, Gln157, Phe184, Lys185, or Tyr186 codon of AtPCS1. All oligonucleotides used are shown in Table S1. The oligonucleotide primers and the complementary nucleotides were incorporated to generate a mutated pET28b-AtPCS. The polymerase chain reaction (PCR) products were then treated with *Dpn* I to digest the parental DNA template, and the mutation-containing synthesized DNA was transformed into *Escherichia coli* DH5α cells (Sigma-Aldrich). Mutagenesis was confirmed by sequencing the coding sequence in all cases. The recombinant proteins of the single mutants were then produced and purified.

### Recombinant protein expression of AtPCS1 variants

The AtPCS1 cDNA was amplified by PCR and cloned to a pET28b vector (Novagen) as an in-frame fusion with a hexahistidine tag on the N-terminus as described previously [28]. The plasmids were transformed into *E. coli* BL21 (DE3) cells (Sigma-Aldrich) and selected on kanamycin plates. Protein expression was induced by adding 1 mM isopropyl-β-D-thiogalactopyranoside (IPTG) and culturing at 30°C for 4.5 h. The bacterial cells were than harvested and suspended in a lysis buffer (100 mM Tris-HCl, pH 8.0, 300 mM NaCl, 1 mM dithiothreitol, 1 mM β-mercaptoethanol with a protease inhibitor cocktail, Roche) and disrupted using sonication (S3000, Misonix). The lysate was centrifuged for 30 min at 15,000 rpm (Beckman Avanti J25, rotor JA25.5). The supernatant was collected and mixed with Ni Sepharose 6 Fast Flow (GE Healthcare) equilibrated in 100 mM Tris-HCl, pH 8.0, 300 mM NaCl, and 10% glycerol. After washing with an equilibration buffer containing 20 mM imidazole, the recombinant AtPCS1 was eluted with 200 mM imidazole. The protein fractions were incubated with 10 mM EGTA for 1 h prior to concentration and desalting by Amicon Ultra (10,000 MW cutoff, Millipore). Protein concentration was determined using the dye-binding Bradford method [34] and the microassay system from a Protein Assay Kit (Bio-Rad), using bovine serum albumin as the protein standard. The composition and purity of the protein fractions were assessed using 12.5% sodium dodecyl sulfate polyacrylamide gel electrophoresis (SDS-PAGE) in Figure S1.

### Measurement of PC and PCS activity

Assays of the standard activity of PCS were performed at 37°C for 10 min in 1 mL reaction mixtures containing 10 μg of AtPCS1, 200 mM HEPES-NaOH (pH 8.0), 10 mM GSH, and 50 μM CdCl_2_. The reactions using 10 mM *S*-methylglutathione were performed in the same conditions, except Cd was omitted from the reaction medium. The incubation times of the Q50A, Q50N, C56A, Q157N, F184A, Y186A, and R152/K185 variants were extended to 180 min to ensure accumulation of sufficient product for high-performance liquid chromatography (HPLC) analysis. All reactions were blocked by the addition of trifluoroacetic acid at a final concentration of 5% (v/v) and stored at -80°C until reverse phase (RP)-HPLC analysis. Aliquots (50 μL) from individual reaction mixtures were analyzed using HPLC with a reverse-phase column (LiChrospher 100 RP-18e, Merck). A linear concentration gradient (0%–20%) of acetonitrile, containing 0.05% trifluoroacetic acid, was used for the elution of PC, which was measured and converted into moles of GSH by comparing with the standard GSH peak. The PCS activity was defined as the GSH molar equivalent of PC_2_, PC_3_, and PC_4_ produced per mole of enzyme per minute as described previously [28]. 

### Equilibrium dialysis of AtPCS1

The methods applied were those of Vatamaniuk et al. [20], with modifications. The dialysis buffer (10 mM Tris-HCl, pH 8.0) contained 10 μM CdCl_2_ to provide the saturation level of Cd-bound AtPCS1. The AtPCS1 (0.36 μM) was dialyzed against 100-fold volumes of the buffer at 4°C for 12 h on a dialysis membrane (14,000 MW cutoff, EIDIA). The protein-bound Cd^2+^ was estimated by measuring the concentrations of Cd in the bulk medium outside the dialysis bag, and in the solution within the dialysis bag. A flame atomic absorption spectrophotometer (932 plus, GBC) was used to measure Cd at 228.8 nm. 

### The kinetics of AtPCS1-catalyzed PC_2_ synthesis

The PCs with varying degrees of polymerization catalyzed by AtPCS1 and its mutants were measured at 37°C in 1 mL reaction mixtures containing 200 mM HEPES-NaOH (pH 8.0), 1 μg AtPCS1, 100 μM CdCl_2_, and 10 mM GSH. The initial velocity of PC_2_ formation was then determined (Figure S2). The incubation time for kinetics analysis was sufficiently short to ensure exclusive synthesis of PC_2_, which precluded the synthesis of the longer chain PC. The limiting reaction time of wild-type AtPCS1, S51A, and Y55W was 2 min, whereas that of E52D was 3 min to ensure the accumulation of sufficient product for HPLC analysis. The assays were performed at 37°C in 1 mL reaction mixtures containing 0.5 μg PCS, 200 mM HEPES-NaOH (pH 8.0), and the indicated concentrations of GSH and CdCl_2_. The kinetic parameters were analyzed using the SigmaPlot Enzyme Kinetics Module and determined from the Michaelis-Menten and Lineweaver-Burk plots (Figure S3).

## Results

### A proposed binding cavity for the second substrate in proximity to the active site of AtPCS1

**Figure 1:**
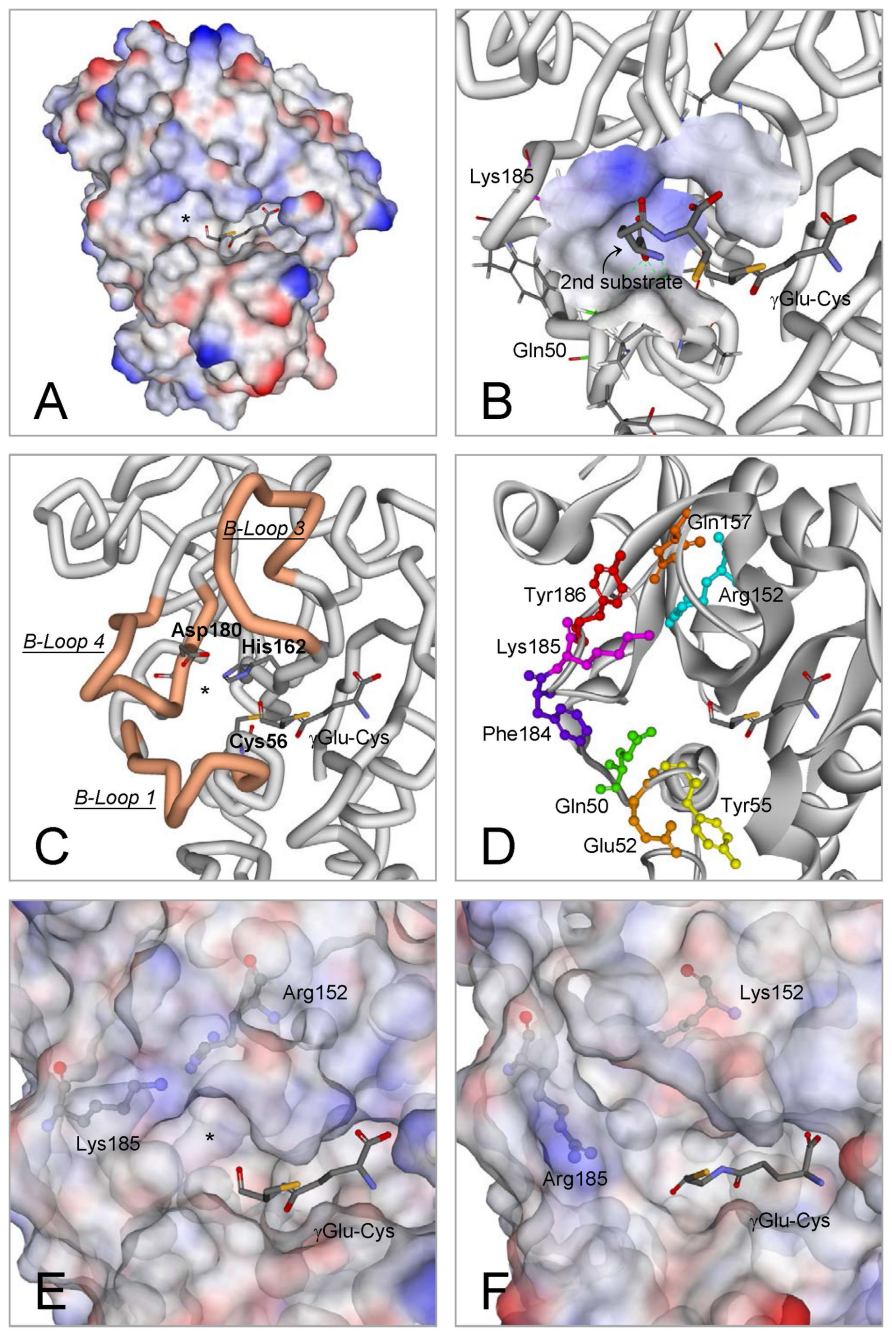
Computer simulation of the structure and active site of AtPCS1. (**A**) An overview of the molecular model of AtPCS1, with the putative second substrate binding cavity indicated with an asterisk. (**B**) Docking prediction for the second substrate (γGlu-Cys) and its binding cavity. The receptor interface is colored according to its ionizability. Positively and negatively charged surfaces are individually indicated by blue and red, respectively. (**C**) The cavity is enclosed by 3 loops (B-loop 1, B-loop 3, and B-loop 4). The catalytic triad (Cys56, His162, and Asp180) is also shown. (**D**) Six conserved residues involved in the formation of the cavity (Gln50, Arg152, Gln157, Phe184, Lys185, and Tyr186), and 2 partially conserved residues on loop 1 (Glu52 and Tyr55). (**E** and **F**) The molecular surfaces of the wild-type AtPCS1 and the R152K-K185R double mutant are displayed for comparison, and the asterisk indicates the putative second substrate binding cavity. The structures were predicted using the coordinates of 2BU3 as a template in Discovery Studio 3.0 (Accelrys). The molecular models were subjected to energy minimization and validation by using Protein Health in Discovery Studio.

**Figure 2:**
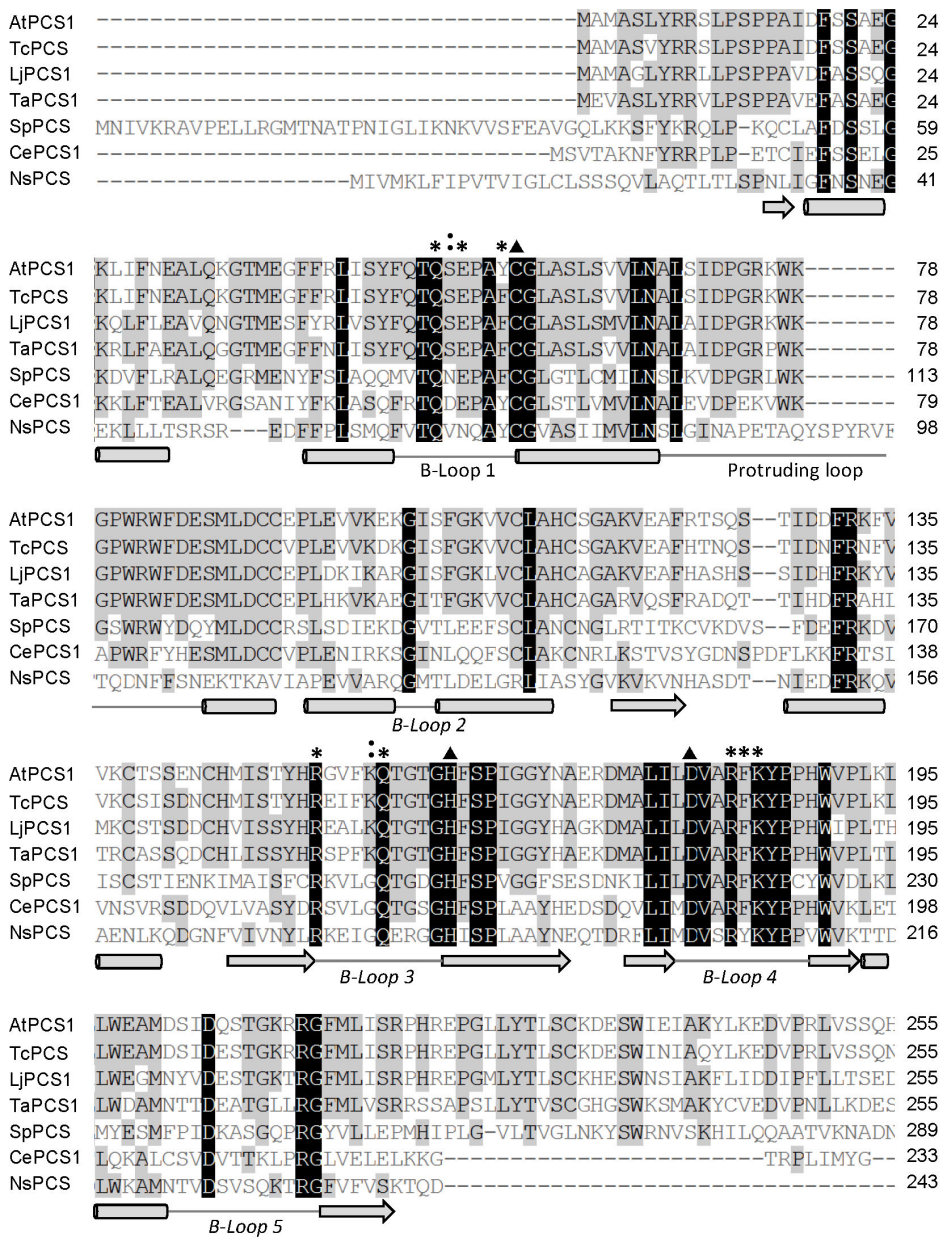
Sequence alignment of the N-terminal domain of AtPCS1 with eukaryotic PCS sequences. The black and gray boxes show the consensus residues on PCS. The black boxes indicate identical amino acids, and the gray boxes indicate highly conserved sequences. The triangles denote the positions of the catalytic triad. The asterisks indicate the amino acids at Gln50, Glu52, Tyr55, Arg152, Gln157, Phe184, Lys185, and Tyr186 on AtPCS1, and the colons indicate Ser51 and Lys156. The cylinders indicate α-helixes, the arrows indicate β-sheets, and the lines indicate loops. The sequences were aligned using Vector NTI (Invitrogen). At, *Arabidospis thaliana*; Tc, *Thlaspi caerulescens*; Lj*, Lotus japonicus*; Ta*, Triticum aestivum*; Sp*, Schizosaccharomyces pombe*; Ce*, Caenorhabditis elegans*; Ns, Nostoc.

In our previous study, using the crystal structure of the NsPCS γ-Glu-Cys acyl-enzyme intermediate (2BU3) [31] as the template, we predicted the protein structure of the N-terminal domain of AtPCS1 (residues 12–218) [28]. We did not include the C-terminal domain in the AtPCS1 model because native NsPCS contains only the N-terminal half of the eukaryotic PCS (Figure 1A). Vivares et al. proposed that a cavity in proximity to the active site of NsPCS might represent the second substrate binding site [31]. Our simulated AtPCS1 model showed a similar cavity (indicated by an asterisk in Figure 1A), surrounded by the 3 loops denoted as B-loop 1, B-loop 3, and B-loop 4, according to the nomenclature for the structure of NsPCS (Figure 1C). Vivares et al. also described that the second substrate binding cavity is shaped and surrounded by several key amino acids on the B-loops: Gln64, Arg173, Lys206, and Tyr207 [31]. Our results indicated that these residues were strictly conserved among the PCS sequences (Figure 2), and equivalent to the amino acids at Gln50, Arg152, Lys185, and Tyr186 on AtPCS1 (Figure 1D). 

To evaluate the possible roles of these amino acids in the catalytic mechanism, we generated recombinant proteins with mutations in the positions of the amino acids, and used them in structure-function studies. In addition to the mentioned residues, we selected several other conserved amino acids on the B-loops as targets for the mutation experiments: Glu52 and Tyr55 on B-loop 1, Gln157 on B-loop 3, and Phe184 on B-loop 4. As shown in Figure 1D, we evaluated the roles of 2 basic (Arg152 and Lys185), one acidic (Glu52), 3 aromatic (Tyr55, Phe184, and Tyr186), and 2 amide residue-containing (Gln50 and Gln157) amino acids. 

### Consensus amino acid residues on the B-loops surrounding the cavity are crucial for the catalytic activity of AtPCS1

Among the 8 consensus amino acids involved in the formation of the second substrate binding cavity, we hypothesized that Arg152 and Lys185 contribute positive charge interactions on the nonpolar surface of the cavity (Figure 1E). The catalytic activity of AtPCS1 was reduced significantly following the replacement of these 2 residues by Ala (Table 1). Mutants without a change in the status of charge distribution, in particular the R152K and K185R mutants, also showed significantly reduced AtPCS1 activity. Mutants with 2 substitutions in R152 and K185, including R152A-K185A and R152K-K185R, showed fully abrogated AtPCS1 activity. We observed similar results following single or double mutations in these positions on His (Table 1). These observations indicated that the positive charge and the locations of these 2 residues are essential for catalytic activity. The simulated structure of the second substrate cavity for the double mutant R152K-K185R (Figure 1F) showed a variant conformation from the wild type (Figure 1E). It is likely that this cavity on the mutants might not be able to accurately recognize the second substrate. 

**Table 1:** Activity of the AtPCS1 mutants at Arg152 and Lys185.

Substitution	AtPCS1 & mutants	Relative activity (%)
None	**Wild type**	**100**
Alanine	R152A	0.02
	K185A	0.02
	R152A-K185A	N.D.
Lysine or Arginine	R152K	0.01
	K185R	0.56
	R152K-K185R	N.D.
Histidine	R152H	0.25
	K185H	0.01
	R152H-K185H	N.D.

The standard assay of the activity of the wild-type PCS was performed at 37°C for 10 min in 100 μL of the reaction mixture. The incubation times for the Arg152 and Lys185 mutants were extended to 180 min to ensure accumulation of sufficient product for HPLC analysis. Data are presented as mean ± standard error of the mean (SE) of 6 replicates from 2 independent experiments. All standard deviations were less than 0.01. ND = undetectable.

**Figure 3:**
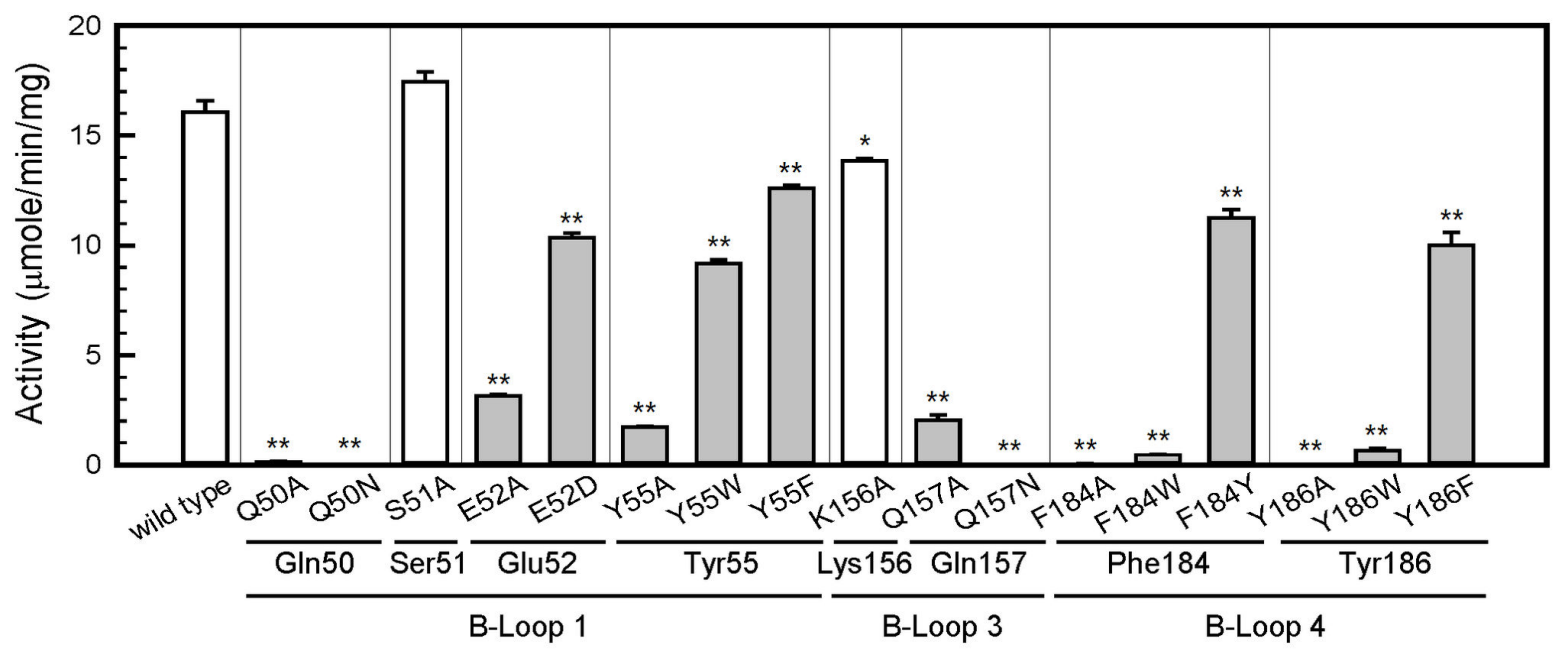
The consensus residues on B-loops play critical roles in AtPCS1 activity. The PCS activities of the single substitutions of the conserved residues on B-loop 1, B-loop 3, and B-loop 4 were compared. Substitutions of these conserved amino acids affected PCS activity to various extents (gray bars). Two nonconserved residues (Ser51 and Lys156) unrelated to the formation of the cavity were included for comparison (open bars). Data are presented as mean ± SE from 3 replicates, and the asterisk indicates a significant difference between wild type and mutants based on analysis of variance (ANOVA) and the Student *t*-test (*, *P* < 0.01; **, *P* < 0.001).

We also generated recombinant proteins with mutations to evaluate the roles of the conserved residues on B-loop 1 (Gln50, Glu52, and Tyr55), B-loop 3 (Gln157), and B-loop 4 (Phe184 and Tyr186). First, we changed these residues to Ala. As shown in Figure 3, the mutants Q50A, E52A, Y55A, Q157A, F184A, and Y186A all showed very low AtPCS1 activity. We included 2 nonconserved amino acids in the mutation experiments for comparison (S51A and K156A), and both showed normal AtPCS1 activity. We then investigated the roles of these conserved residues using amino acids with similar characteristics. E52D showed comparable activity to E52A, indicating that the negative charge at this position plays only a minor role in catalysis. The activities of Y55W and Y55F were approximately two-thirds of the overall catalytic activity of the wild type. Our results indicated that the steric effect is crucial in Phe184 and Tyr186, because both F184W and Y186W showed only 4% of the overall activity, whereas the F184Y and Y186F mutants retained 60% of the activity compared with the wild type. It is likely that the larger size of the indole group on Trp, which might occupy additional space in the cavity, interferes with the binding of the second substrate. The Q50N and Q157N mutants showed complete loss of function, indicating that the chain length in these 2 positions is crucial for catalytic activity. Overall, our results indicate that in addition to the chemical characteristics of the consensus amino acid residues, the sizes of the side chains are crucial for the appropriate formation of the second substrate binding cavity. 

### Tyr55 is the putative Cd-binding site on the second substrate Cd∙GS_2_

To further investigate the amino acid residues on AtPCS1 that are involved in the binding of the second substrate, we performed computer docking using Discovery Studio (Figure 1B). Our efforts to dock the “first γGlu-Cys”-occupied AtPCS1 with another GSH molecule were unsuccessful because of limited space in the putative cavity for the second substrate. It was most likely that the molecule did not enter the second binding cavity entirely. Thus, the cavity could only bind γGlu-Cys, which has a smaller molecular size than GSH. The docking simulation indicated that the second substrate probably enters the cavity through its amino terminus (Figure 1B). The alpha carbon of the N-terminal Glu moiety of GSH has one amino group and one carboxyl group. The positive electric cluster formed by Lys185 and Gln50 might attract the carboxyl group. This interaction leaves the free amino group of the second substrate available to attack the acylated γGlu-Cys on the active site and produce PC.

**Figure 4:**
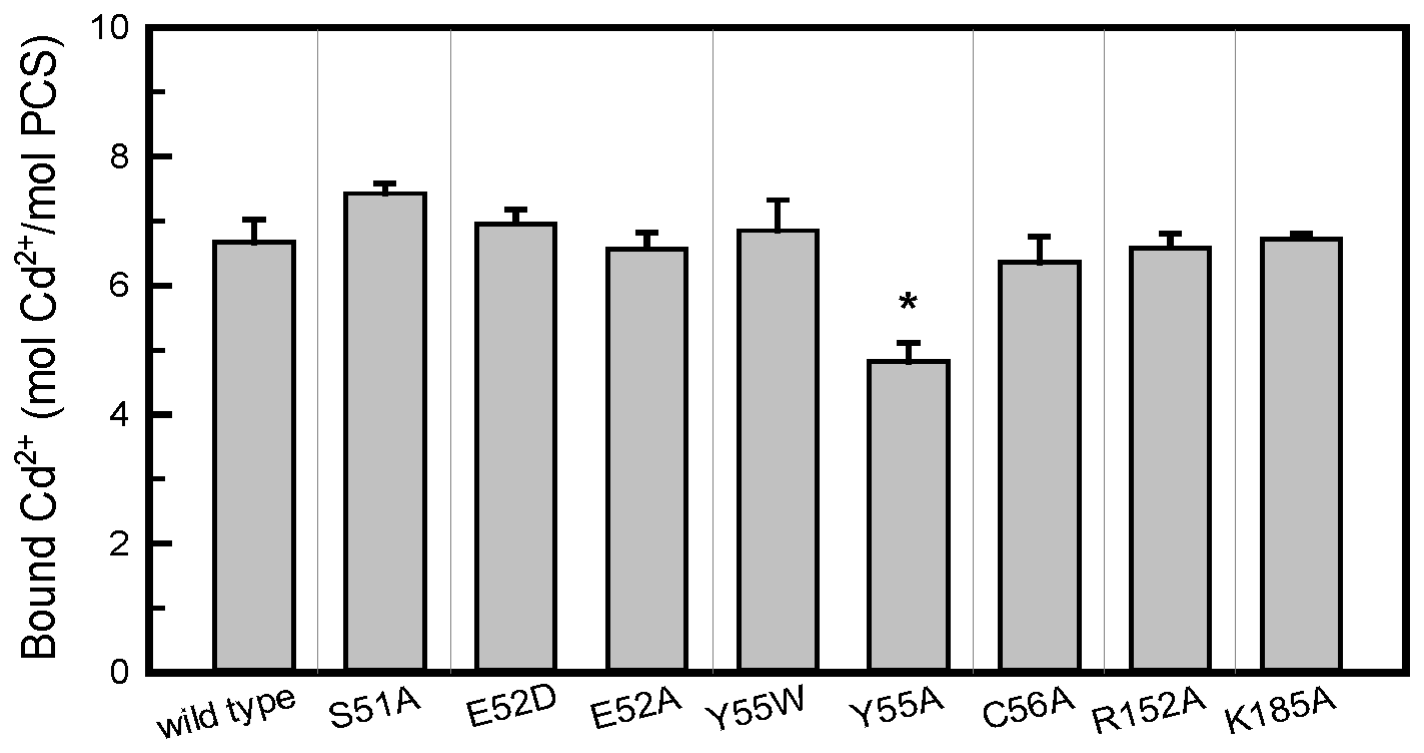
Mutation at Tyr55 might reduce the Cd binding capacity of AtPCS1. The Cd binding capacities of AtPCS1 and its mutants were analyzed using equilibrium dialysis. The samples were dialyzed against Tris-HCl buffer (pH 8) containing 10 μM CdCl_2_ at 4°C for 12 h. Data are presented as mean ± SE from 3 replicates, and the asterisk indicates a significant difference based on ANOVA and the Student *t*-test (*, *P* < 0.01).

However, the thiol group of the Cys residue on the second GSH might interact with several amino acid residues on the B-loop 1 by forming a bridge through the Cd ion. Two potential candidates are Glu52 and Tyr55. The carboxyl group on Glu52 might couple with the cationic Cd through ionic interaction, whereas the aromatic group on Tyr55 might produce a cation-π complex with Cd [35,36]. We identified that Tyr55 showed the capacity to bind Cd, because the total number of Cd ions bound to the Y55A mutant reduced from 7 to 5 (Figure 4). We also observed that the Y55W retained its full Cd binding capacity, which might be contributed by the cation-π interaction. Mutations at Gln52 to Asp (E52D) or Ala (E52A) had no effects on Cd binding capacity (Figure 4). Maier et al. proposed that a fragment on SpPCS or TaPCS1 corresponding to Tyr55-Cys56 of the AtPCS1 serves as a Cd binding motif [24]. However, we observed that the AtPCS1 with a mutation at Cys56 (C56A) retained the same Cd binding potential as the wild-type AtPCS1 (Figure 4). 

Because the Y55W mutant retained approximately 60% of the catalytic activity of the wild-type AtPCS1 (Figure 3), we used it to measure the Michaelis-Menten constants (*K*_m_) for the first (GSH) and second (Cd∙GS_2_) substrates. We compared the kinetic parameters of the Y55W with those of the wild-type AtPCS1 and the 2 mutants, S51A and E52D. As shown in Table 2, the S51A and Y55W showed minimal changes in their *K*_m(GSH)_, whereas that of the E52D showed a 2-fold increase in *K*_m(GSH)_ compared with the wild type. However, the affinity of the Y55W to its second substrate was reduced significantly because its *K*_m(Cd∙GS2)_ was nearly 3-fold higher than those of the other AtPCS1 variants. In this mutant, the change of the hydroxyl-phenyl group to an indole group might have interfered with the binding affinity of the second substrate with the cavity. 

**Table 2:** Kinetic parameters for the synthesis of PC_2_ by AtPCS1 and its mutants.

AtPCS1 & mutants	*V*_max_ (μmole/mg/min)	*K*_m(GSH)_ (mM)	*K*_m(Cd∙GS2)_ (μM)	*V*_max_ / *K*_m(GSH)_	*V*_max_/ *K*_m(Cd∙GS2)_
Wild type	227 ± 13	18.3 ± 1.6	3.58 ± 0.32	12.4	63.4
S51A	245 ± 9	13.9 ± 0.8	5.74 ± 0.28	17.6	42.7
E52D	259 ± 16	36.1 ± 2.8	3.19 ± 0.28	7.2	81.2
Y55W	272 ± 13	25.8 ± 1.7	10.3 ± 0.6	10.5	26.3

The kinetic data were analyzed using the SigmaPlot Enzyme Kinetics Module.

**Figure 5:**
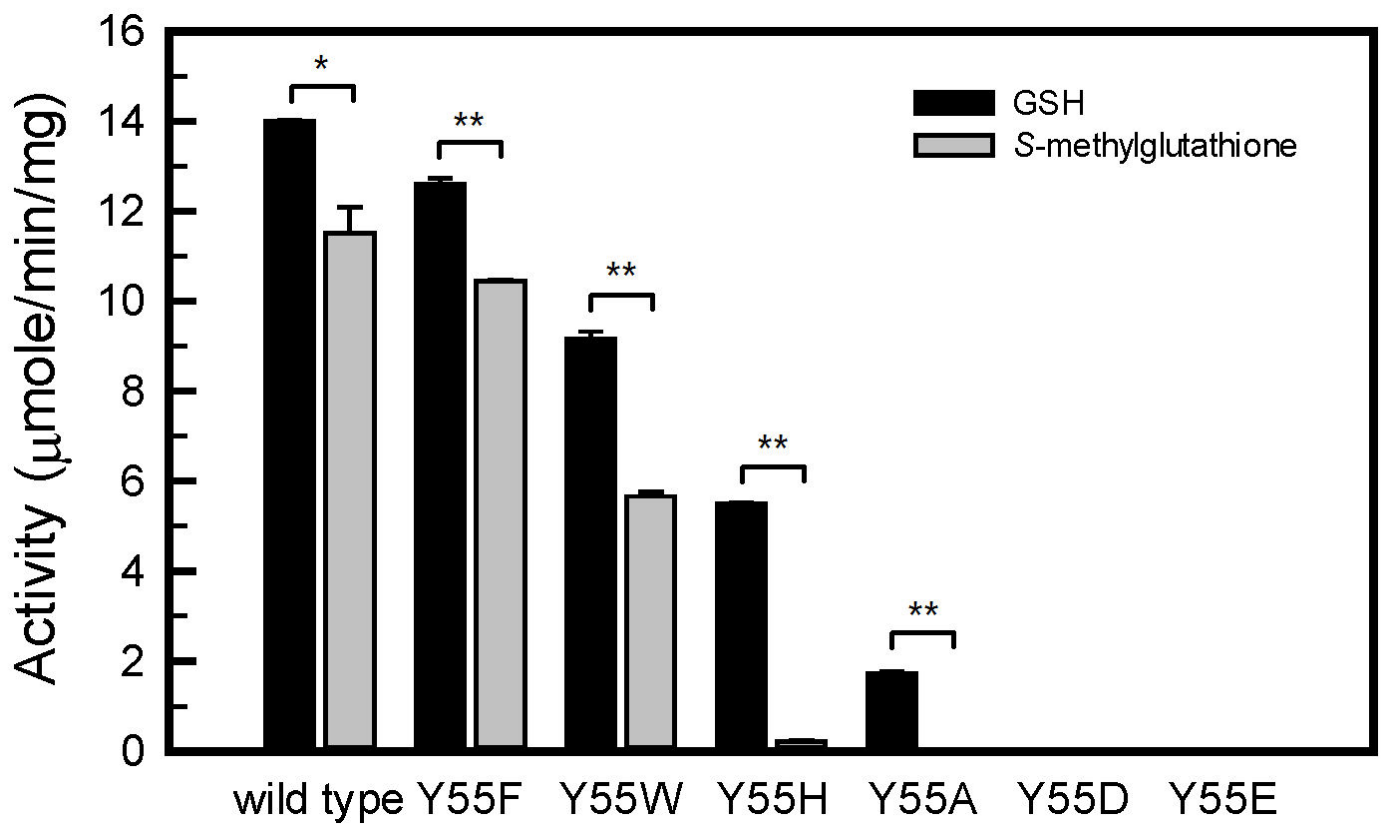
Comparison of AtPCS1 activity among Tyr55 mutants using GSH or *S*-methylglutathione as substrates. The PC synthesis activity was compared among Tyr55 mutants using GSH or *S*-methylglutathione as substrates. The standard PCS assay (using GSH) was performed as described in “Materials and Methods.” The assay using *S*-methylglutathione as the substrate was performed in the same conditions except no Cd was added to the reaction. Data are presented as mean ± SE from 3 replicates, and the asterisk indicates a significant difference between wild type and mutants based on ANOVA and the Student *t*-test (*, *P* < 0.01; ** *P* < 0.001).

To further evaluate the role of the side chain of Tyr55, we changed Tyr55 to His, Asp, or Glu and compared the catalytic behaviors of the Tyr55 variants using an alternative substrate containing a blocked thiol group, *S*-methylglutathione, and GSH (Figure 5). In the reactions using GSH as the substrate with Cd supplied, the PCS activities of the mutants was in the order of wild type > Y55F > Y55W > Y55H Y55A > Y55D = Y55E (Y55D and Y55E had undetectable activity). Mutants with aromatic rings in place of Tyr55 retained some of their catalytic activity. However, the mutants with negatively charged residues replacing Tyr55 were absent of activity (Y55D and Y55E). In a parallel experiment, we used *S*-methylglutathione as the catalytic substrate to produce PC analogues in the absence of Cd. Our results were similar to those obtained using GSH for the mutants except in the case of Y55H. This mutant retained approximately one-third of its AtPCS1 activity in the normal reaction; however, it showed almost no activity using *S*-methylglutathione as a substrate. It is likely that the aromatic imidazole ring in His can bind to Cd, but not to the *S*-methyl group. 

## Discussion

**Figure 6:**
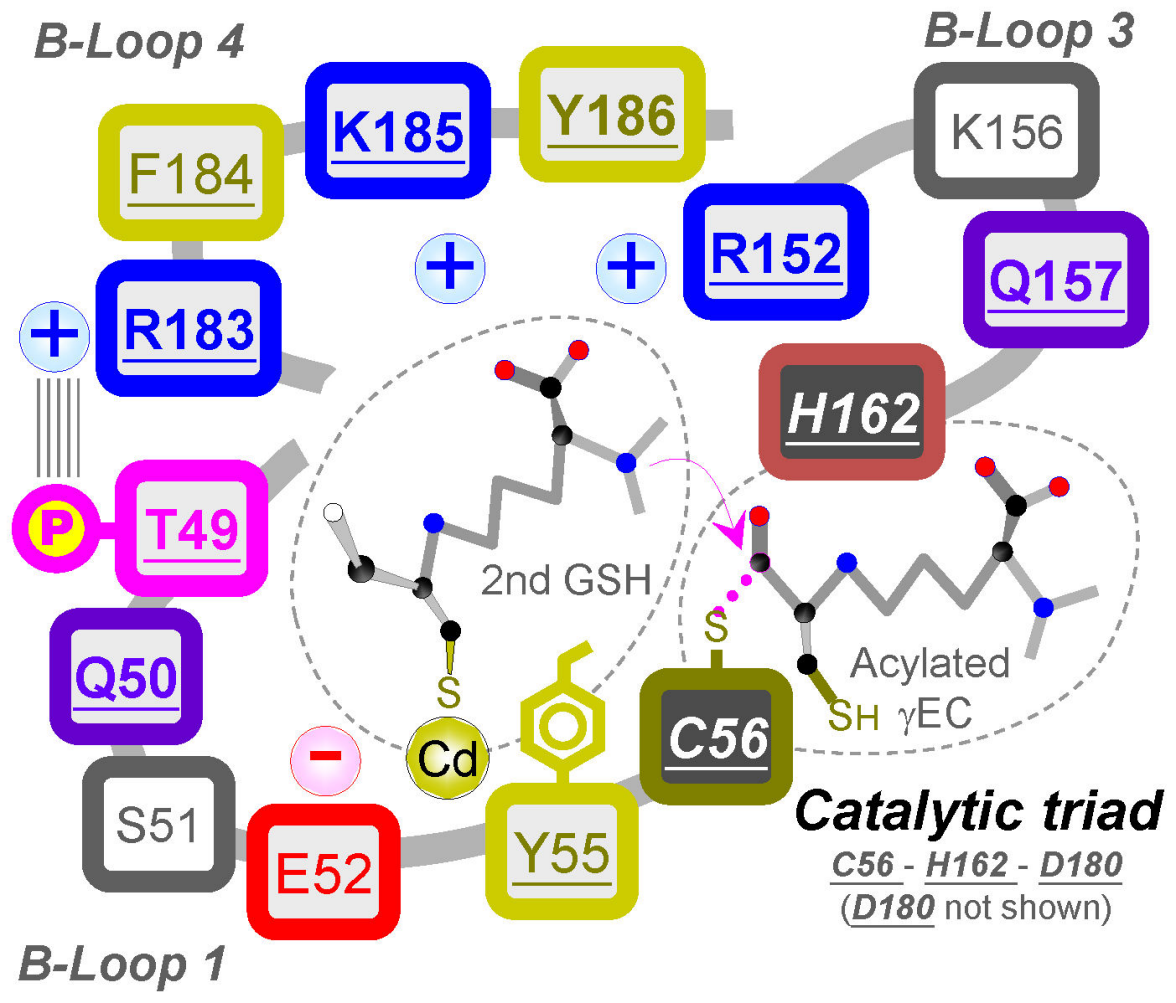
A schematic representation of the AtPCS1 active site

Previous studies have described that the catalytic reaction of PCS occurs through a ping-pong mechanism [20,30]. The mechanism uses 2 molecules of GSH as substrates to produce the shortest phytochelatin, PC_2_. The first GSH is acylated to Cys56 at the active site, which releases the Gly moiety in the C-terminus of GSH. The second GSH then enters the second substrate cavity in a complex form of Cd∙GS_2_. Once attached to the cavity, a free GSH is released from the complex [20]. The N-terminal amino group of the bound GSH then attacks the acylated γGlu-Cys to produce the PC. This catalytic mechanism includes 2 binding sites: one for the first GSH, the other for the second GSH and Cd. Figure 6 shows a schematic representation of the AtPCS1 active site, including the catalytic triad, the tentative second binding cavity and its surrounding 3 B-loops, and 2 substrates. In this study, we investigated the second substrate binding cavity and the manner in which the second GSH binds to the cavity.

The second substrate binding cavity is surrounded by 3 loop structures in proximity to the active site of PCS. Several amino acids on these loops are consensus among PCS sequences from various species (Figure 2). Site-directed mutagenesis studies of these amino acids provided further evidence of the process for the binding of the second GSH and Cd to the cavity. The second GSH might enter the cavity through the N-terminus that contains a carboxyl and an amino group. Our findings indicated that the positive charge in the cavity might recognize the carboxyl group, predominantly through the amino acid at Lys185, leaving the amino group available to attack the carbonyl carbon of the acylated γGlu-Cys at Cys56 of the active site. The thiol group of Cys on the second GSH might bind to Tyr55 on the enzyme by using Cd as a bridge. It is possible that this Cd is bound to the aromatic residue of Tyr55 through the cation-π interaction, because the AtPCS1 Cd-binding capacity reduced with Tyr55 mutation to Ala (Figure 4), and the Y55W mutant retained its full Cd-binding capacity, probably because of the high affinity of the metal ion for the indole group of Trp [37]. However, our kinetic studies indicated that Y55W had lower affinity for the second substrate, Cd∙GS_2_ (Table 2). Although the indole group contributes substantially to the binding of Cd ion, its bulky size might interfere with the formation of an appropriate conformation for the second substrate. 

*S*-methylglutathione is an alternative substrate for PCS. PCS efficiently uses *S*-methylglutathione to produce PC analogues in the absence of Cd [20,25]. The side chains of Tyr, Phe, and Trp are amphipathic and capable of forming nonpolar interactions with a methyl group [38,39]; therefore, it is possible that the methyl group on this analogue might replace Cd. These characteristics might explain our observations that all Tyr55 mutations to other aromatic residues (Y55F, Y55W, and Y55H) were associated with a range of activities toward GSH in the presence of Cd (Figure 5). However, Y55H could not use *S*-methylglutathione as a substrate, possibly because of poor hydrophobic interaction between the imidazole residue and the methyl group on *S*-methylglutathione [38]. 

Our results also indicated that all the consensus amino acids on the B-loops are essential for the enzymatic activity of AtPCS1. Among them, Arg152 and Lys185 are 2 basic amino acids that might play roles in interaction with the second GSH. Their mutants with Ala replacing the charged residues showed very low activity (Table 1). Mutations from Arg to Lys (R152K), or Lys to Arg (K185R), were associated with complete activity loss. The simulated model for AtPCS1 and our docking results showed that Lys185 might directly interact with the second substrate, whereas Arg152 stabilizes the binding site structure through noncovalent bonds with residues His162, Asp180, and Tyr186 (Figure S4). Our experiments using Q157N indicated that Gln157 had critical effects on chain length (Figure 3). If we changed the small aromatic amino acids at Phe184 and Tyr186 to Trp, the mutants (F184W and Y186W) showed markedly reduced activity (Figure 3). These results indicated that the charge or polarity of amino acid residues is not the only factor to affect PCS catalysis. The sizes and shapes of the side chains on the consensus amino acids surrounding the second substrate binding cavity are critical for the formation of an appropriate conformation for the incoming substrate. However, it was also possible that these mutations affect the substrate binding in the first site, which is close to the putative second binding site. Thus, we could not exclude the possibility that the reduced PCS activities might result from indirect modifications of the first substrate binding site. 

It is interesting to note that Q50N completely loss catalytic activity (Figure 3). This residue is corresponding to the Gln19 of papain and Gln64 of NsPCS, where they contribute to the formation of an oxyanion hole. As described by Rea (2012), the oxyanion hole serves to polarize the carbonyl group bound to be broken in the initial nucleophilic attack on the first substrate and stabilize the tetrahedrally distorted transition states of the γ-Glu-Cys donor and enzyme thioester, respectively [27,31]. Therefore, the modification on the chain length at Gln50 was crucial to the catalysis since this residue might participate in the stabilization of the tetrahedral transition state assumed by the first substrate.

In our previous study, we showed that protein kinase phosphorylates Thr49 on AtPCS1, and that phosphorylated AtPCS1 displays significantly increased activity compared to the wild-type AtPCS1 [28]. We hypothesized that phosphorylated Thr49 might interact with the neighboring Arg183 to connect the 2 loop structures and form the catalytic cavity, because Thr49 is located on B-loop 1 and Arg183 is located on B-loop 4 (Figure 6). Both Thr49 and Arg183 are consensus amino acids on PCS (Figure 2). This interaction is triggered by protein phosphorylation and might be critical for the formation of a functional catalytic site. First, the connection of the 2 loops might induce the appropriate change in conformation for the binding of the second GSH. Second, the interaction between these 2 loops might bring the catalytic triad to the appropriate position on the active site, because Cys56 is located on B-loop 1, His is located on B-loop 3, and Asp180 is located on B-loop 4. Third, the connection of the 2 loops might close the cavity, restricting cavity space, and leading to stringent requirements for appropriate amino acid side chains around the cavity. Approximately 66% of the consensus amino acids on AtPCS1 are located on these loops. 

Based on the model of the N-terminal structure of AtPCS1, we gained some information on the tentative second substrate binding site on AtPCS1. It should be bearing in mind that the prokaryotic NsPCS is not an ideal template to model AtPCS1 due to the lower synthetic activity and the lack of the C-terminal domain found in eukaryotic homologs. In this study, the molecular model was useful to guide our experiments in the investigation of the putative second substrate binding site. However, the identification of the second substrate binding site will become clearer when a more accurate model is established.

In conclusion, based on the identification of the consensus amino acids on the loop structures surrounding the active site, molecular modeling, and mutation studies of these amino acids, we propose a possible binding and catalytic mechanism for the second substrate binding cavity on AtPCS1. In combination with the first acylation reaction by the catalytic triad on PCS, our results might elucidate the mechanism underlying the synthesis of PC.

## Supporting Information

Figure S1 **Expression and purification of AtPCS1 and its mutants.** SDS-PAGE (A) and Western Analysis (B) for the soluble fractions from crude extracts of *E. coli* BL21 (DE3) cells expressing His-AtPCS1. Protein (20 μg) was subjected to SDS-PAGE on 12.5% gels, electrotransferred, and probed with anti-His monoclonal antibody (GE healthcare). After purification by Ni-NTA chromatography and dialysis as described in “Materials and Methods”, AtPCS1 (2 μg) was subjected to SDS-PAGE on 12.5% gels (C). The positions of the molecular mass markers are indicated.
(PDF)

Figure S2 **Time course of *invitro* PC synthesis by wild type AtPCS1, S51A, E52D and Y55W.** PC synthesis activity by wild type AtPCS1 (**A**), S51A (**B**), E52D (**C**) and Y55W (**D**) were assayed for the indicated lengths of time and followed by RP-HPLC analysis. PC with varying polymerization (n) values is indicated as follows: PC_2_ (close circle), PC_3_ (open circle) and PC_4_ (close triangle). The PCS activity assay was performed with 10 mM GSH and 100 μM CdCl_2_.
(PDF)

Figure S3 **Kinetic data of PC_2_ synthesis catalyzed by wild type AtPCS1, S51A, E52D and Y55W.** PC_2_ synthesis catalyzed by AtPCS1 in the presence of variable concentration of GSH and CdCl_2_ were shown as Michaelis-Menten plots and the Lineweaver-Burk plots. **A** and **B**, wild type AtPCS1; **C** and **D**, S51A; E and F, E52D; G and H, Y55W.
(PDF)

Figure S4 **Residues involved in the forming of non-covalent bonds with Arg152.** In the simulated structure of AtPCS1, the side chain of Arg152 interacted with surrounding residues by forming hydrogen bonds and cation-π interactions. Besides His162, Asp180 and Tyr186 that are involved in forming the active site structure, Arg152 may be associated with other conserved residues including Tyr150, Phe155, Ser164 and His189.
(PDF)

Table S1 **Oligonucleotides used for site-direct mutagenesis.**
(DOCX)
