# Supplementary figures and images for: Tentative Identification of the Second Substrate Binding Site in Arabidopsis Phytochelatin Synthase
Source: PLoS One. 2013 Dec 5;8(12):e82675. doi: 10.1371/journal.pone.0082675 (PMC3855540; doi:10.1371/journal.pone.0082675)

Coomassie blue

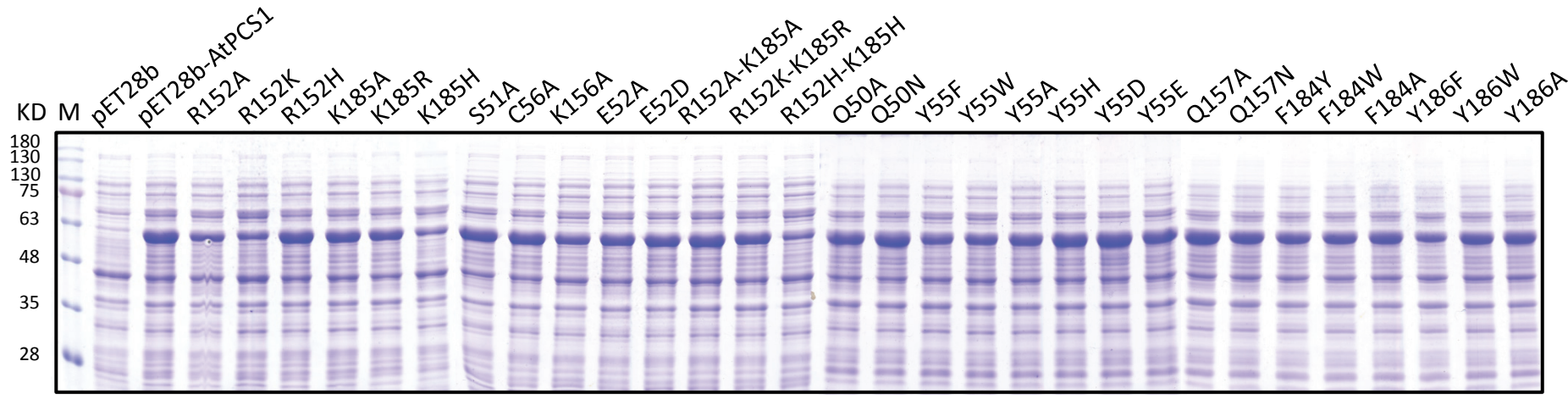

Anti-His tag

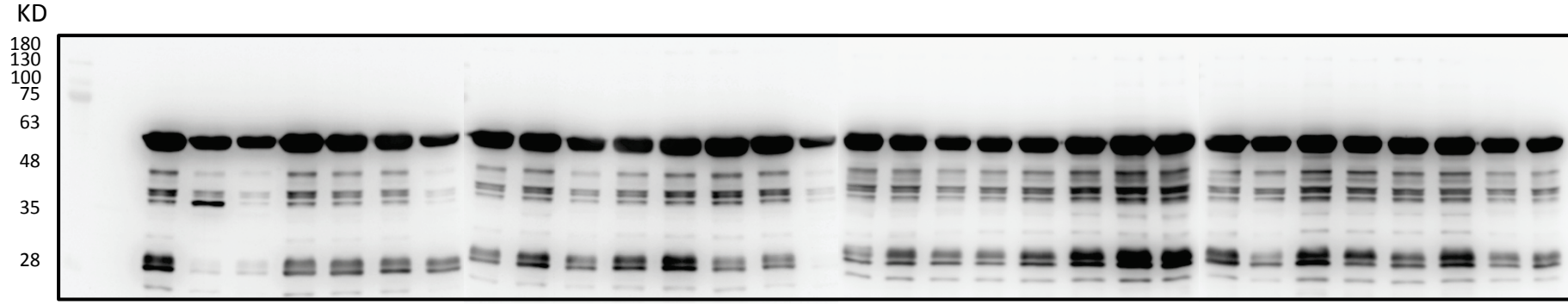

Purified AtPCS1

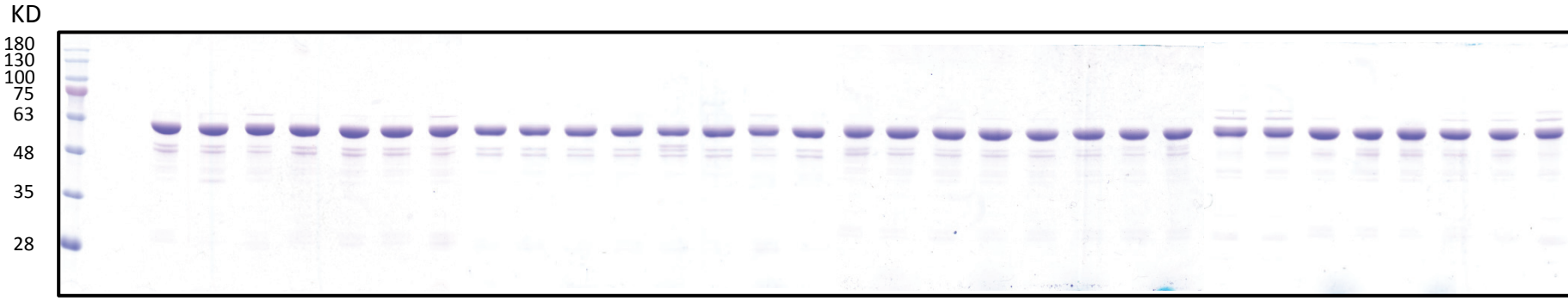

Supplement: Figure S1 — Expression and purification of AtPCS1 and its mutants. SDS-PAGE (A) and Western Analysis (B) for the soluble fractions from crude extracts of E. coli BL21 (DE3) cells expressing His-AtPCS1. Protein (20 μg) was subjected to SDS-PAGE on 12.5% gels, electrotransferred, and probed with anti-His monoclonal antibody (GE healthcare). After purification by Ni-NTA chromatography and dialysis as described in “Materials and Methods”, AtPCS1 (2 μg) was subjected to SDS-PAGE on 12.5% gels (C). The positions of the molecular mass markers are indicated. (PDF) [file pone.0082675.s001.pdf]

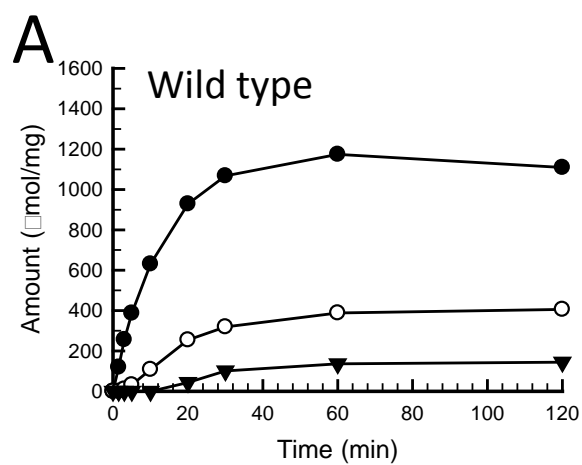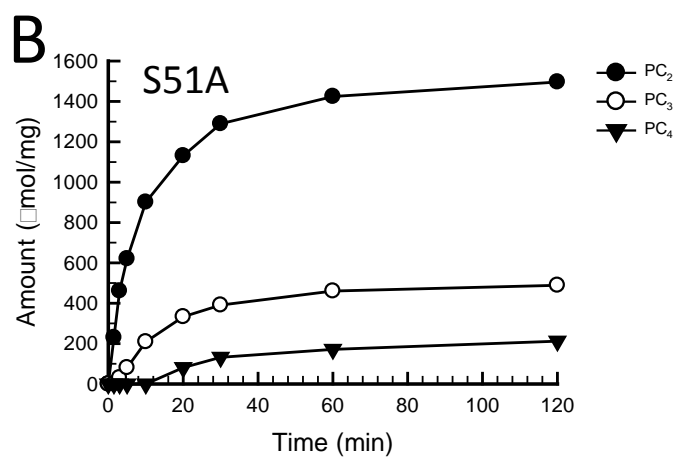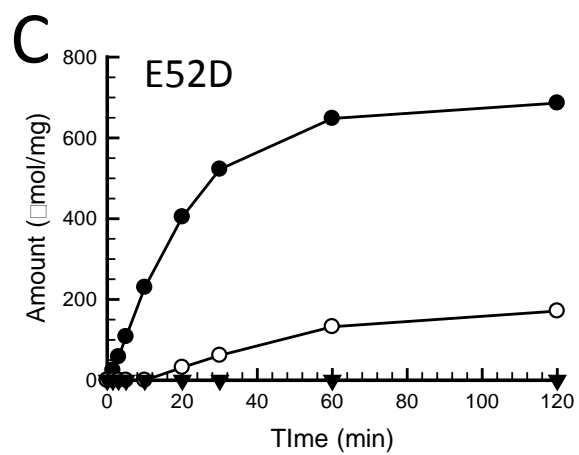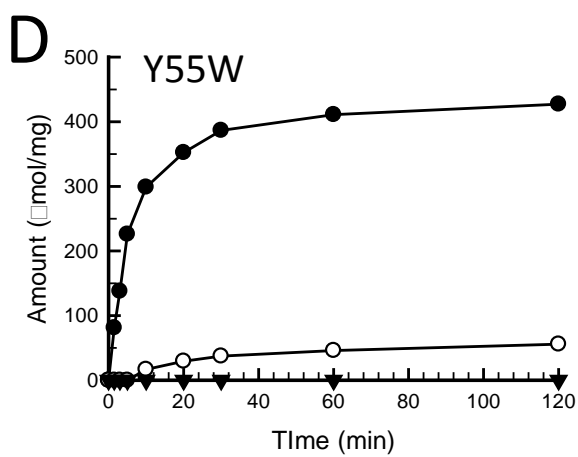

Supplement: Figure S2 — Time course of invitro PC synthesis by wild type AtPCS1, S51A, E52D and Y55W. PC synthesis activity by wild type AtPCS1 (A), S51A (B), E52D (C) and Y55W (D) were assayed for the indicated lengths of time and followed by RP-HPLC analysis. PC with varying polymerization (n) values is indicated as follows: PC2 (close circle), PC3 (open circle) and PC4 (close triangle). The PCS activity assay was performed with 10 mM GSH and 100 μM CdCl2. (PDF) [file pone.0082675.s002.pdf]

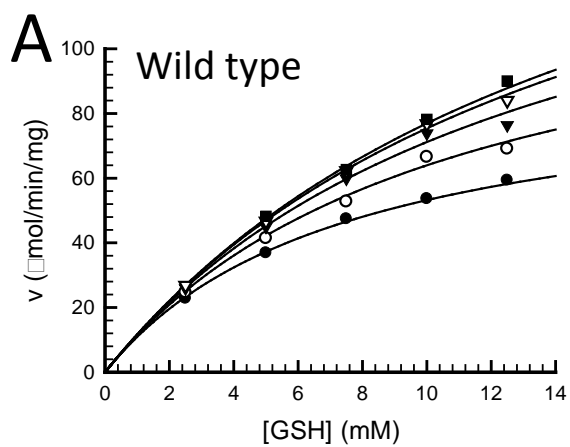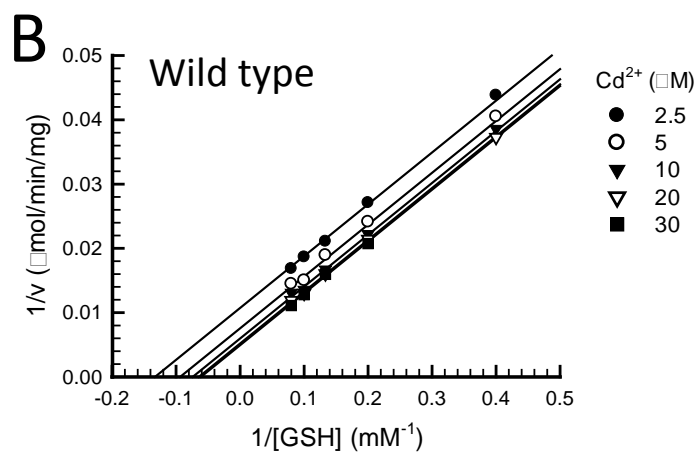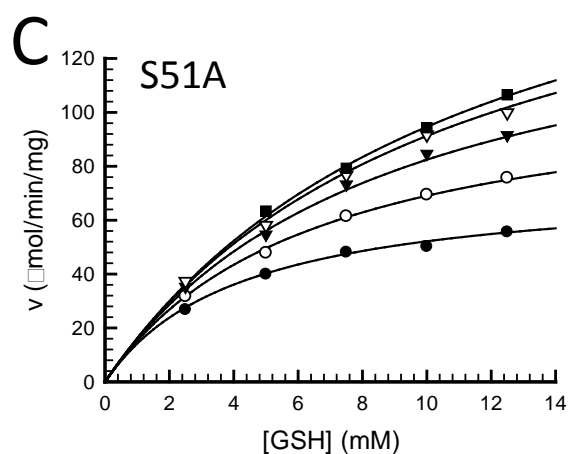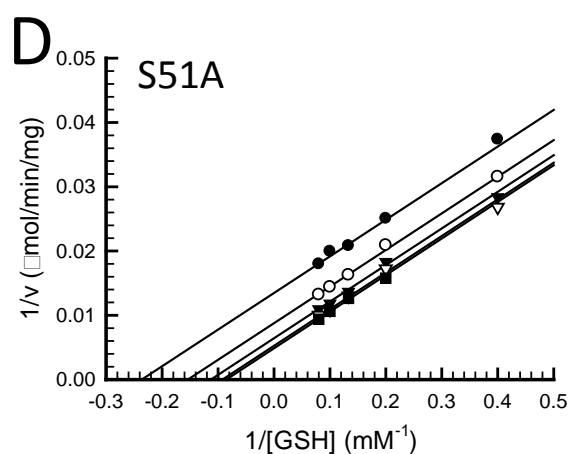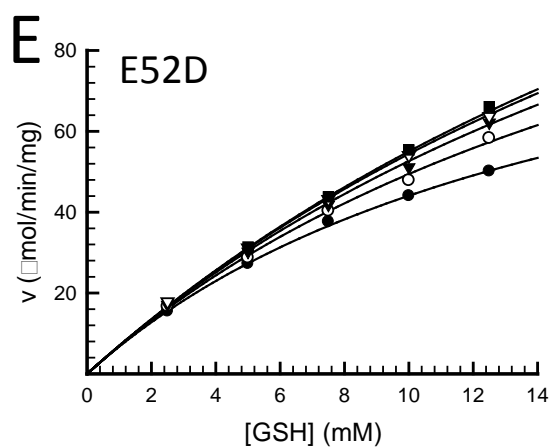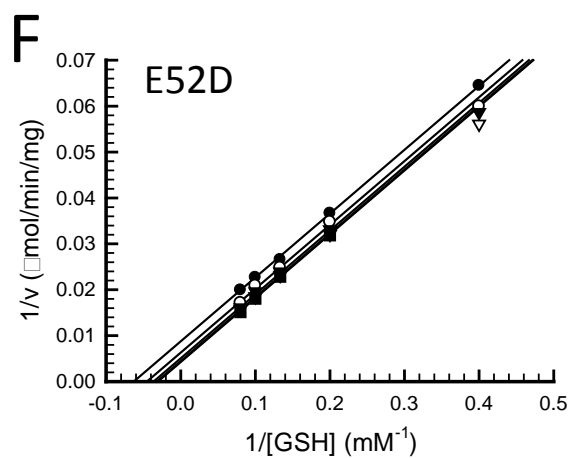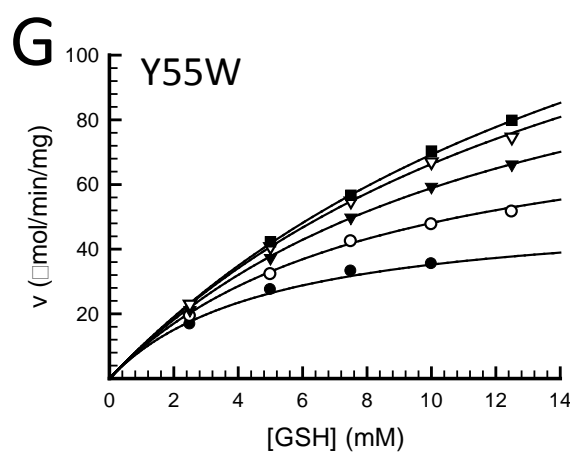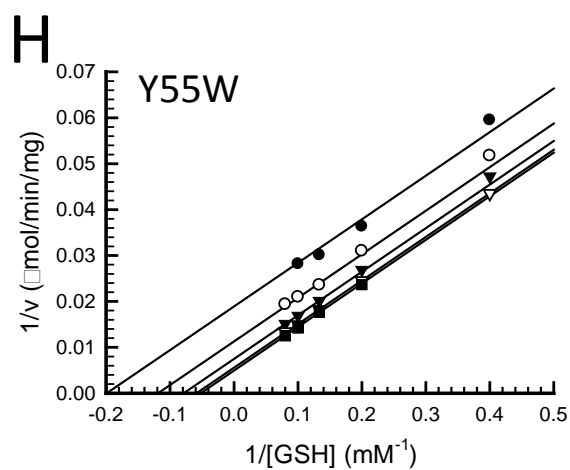

Supplement: Figure S3 — Kinetic data of PC2 synthesis catalyzed by wild type AtPCS1, S51A, E52D and Y55W. PC2 synthesis catalyzed by AtPCS1 in the presence of variable concentration of GSH and CdCl2 were shown as Michaelis-Menten plots and the Lineweaver-Burk plots. A and B, wild type AtPCS1; C and D, S51A; E and F, E52D; G and H, Y55W. (PDF) [file pone.0082675.s003.pdf]

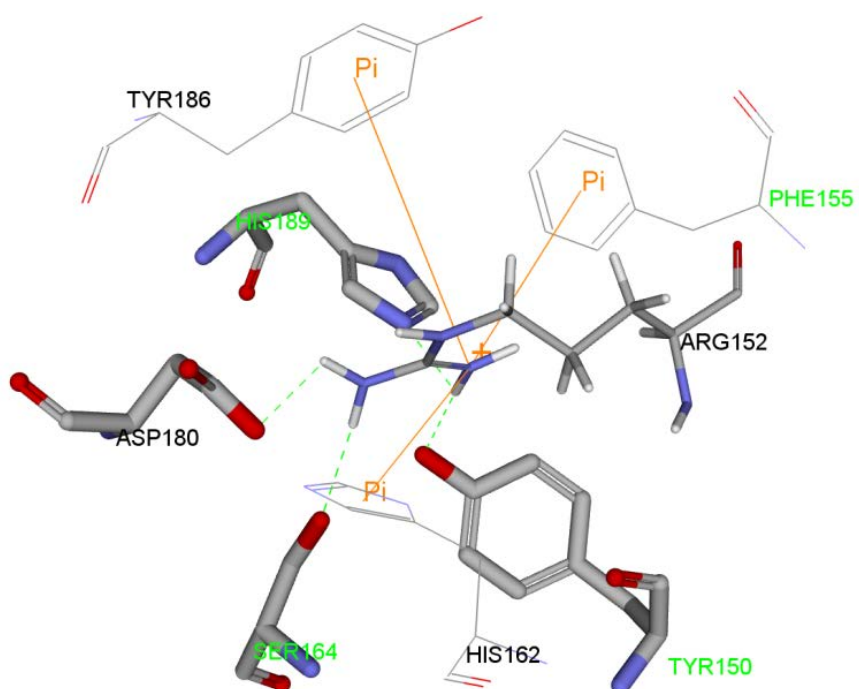

Supplement: Figure S4 — Residues involved in the forming of non-covalent bonds with Arg152. In the simulated structure of AtPCS1, the side chain of Arg152 interacted with surrounding residues by forming hydrogen bonds and cation-π interactions. Besides His162, Asp180 and Tyr186 that are involved in forming the active site structure, Arg152 may be associated with other conserved residues including Tyr150, Phe155, Ser164 and His189. (PDF) [file pone.0082675.s004.pdf]
