# Supplementary material for: Tentative Identification of the Second Substrate Binding Site in Arabidopsis Phytochelatin Synthase
Source: PLoS One. 2013 Dec 5;8(12):e82675. doi: 10.1371/journal.pone.0082675 (PMC3855540; doi:10.1371/journal.pone.0082675)
Supplement: Table S1 — Oligonucleotides used for site-direct mutagenesis. (DOCX) [file pone.0082675.s005.docx]

**Table S1. Oligonucleotides used for site-direct mutagenesis**

| Primers | Sequences (5’-3’) *^a^*^,^ *^b^* |
| --- | --- |
| Q50A | GATTTCGTATTTTCAGACA**GCA**TCCGAACCTGCGTATTGTGG |
| Q50N | GATTTCGTATTTTCAGACA**AAC**TCCGAACCTGCGTATTGTGG |
| S51A | TCGTATTTTCAGACACAA**GCC**GAACCTGCGTATTGTGG |
| E52A | GTATTTTCAGACACAATCC**GCA**CCTGCGTATTGTGGTTTG |
| E52D | GTATTTTCAGACACAATCC**GAT**CCTGCGTATTGTGGTTTG |
| Y55A | CACAATCCGAACCTGCG**GCG**TGTGGTTTGGCTAGTCTC |
| Y55F | CACAATCCGAACCTGCG**TTT**TGTGGTTTGGCTAGTCTC |
| Y55W | CACAATCCGAACCTGCG**TGG**TGTGGTTTGGCTAGTCTC |
| Y55H | CACAATCCGAACCTGCG**CAC**TGTGGTTTGGCTAGTCTC |
| Y55D | CACAATCCGAACCTGCG**GAT**TGTGGTTTGGCTAGTCTC |
| Y55E | GACACAATCCGAACCTGCG**GAA**TGTGGTTTGGCTAGTCTC |
| C56A | CAATCCGAACCTGCGTAT**GCT**GGTTTGGCTAGTCTCTC |
| R152A | CATATGATCTCAACATATCAC**GCG**GGTGTATTTAAGCAGACTGGG |
| R152K | CATATGATCTCAACATATCAC**AAA**GGTGTATTTAAGCAGACTGGG |
| R152H | CATATGATCTCAACATATCAC**CAT**GGTGTATTTAAGCAGACTGGG |
| K156A | CATATCACCGAGGTGTATTT**GCG**CAGACTGGGACTGGTCAC |
| Q157A | TATCACCGAGGTGTATTTAAG**GCG**ACTGGGACTGGTCACT |
| Q157N | TATCACCGAGGTGTATTTAAG**AAT**ACTGGGACTGGTCACT |
| F184A | CTTGATGTTGCTCGT**GCC**AAGTATCCCCCTCACTGGG |
| F184W | CTTGATGTTGCTCGT**TGG**AAGTATCCCCCTCACTGGG |
| F184Y | CTTGATGTTGCTCGT**TAC**AAGTATCCCCCTCACTGGG |
| K185A | CTTGATGTTGCTCGTTTC**GCG**TATCCCCCTCACTGGGG |
| K185R | CTTGATGTTGCTCGTTTC**CGC**TATCCCCCTCACTGGGTTCC |
| K185H | CTTGATGTTGCTCGTTTC**CAT**TATCCCCCTCACTGGGTTCC |
| Y186A | GATGTTGCTCGTTTCAAG**GCT**CCCCCTCACTGGGTTCC |
| Y186W | GATGTTGCTCGTTTCAAG**TGG**CCCCCTCACTGGGTTCC |
| Y186F | GATGTTGCTCGTTTCAAG**TTT**CCCCCTCACTGGGTTCC |

*^a^* The sequences of the complementary nucleotides are not shown.

*^b^* Bold letters indicate the positions of mutated nucleotides.
